# Activation of Host-NLRP3 Inflammasome in Myeloid Cells Dictates Response to Anti-PD-1 Therapy in Metastatic Breast Cancers

**DOI:** 10.3390/ph15050574

**Published:** 2022-05-04

**Authors:** Isak W. Tengesdal, Suzhao Li, Nicholas E. Powers, Makenna May, Charles P. Neff, Leo A. B. Joosten, Carlo Marchetti, Charles A. Dinarello

**Affiliations:** 1 Department of Medicine, Radboud University Medical Center, 6525 Nijmegen, The Netherlands; isak.tengesdal@ucdenver.edu (I.W.T.); leo.joosten@radboudumc.nl (L.A.B.J.); charles.dinarello@cuanschutz.edu (C.A.D.); 2 Department of Medicine, University of Colorado Denver, Aurora, CO 80045, USA; suzhao.li@cuanschutz.edu (S.L.); nicholas.e.powers@cuanschutz.edu (N.E.P.); makenna.may@colorado.edu (M.M.); charles.neff@cuanschutz.edu (C.P.N.)

**Keywords:** NLRP3, IL-1β, anti-PD-1, breast cancer, immunosuppression

## Abstract

Tumor-associated inflammation leads to dysregulated cytokine production that promotes tumor immune evasion and anti-tumor immunity dysfunction. In advanced stage breast cancer, the proinflammatory cytokine IL-1β is overexpressed due to large proportions of activated myeloid cells in the tumor microenvironment (TME). Here, we demonstrate the role of the host nucleotide-binding domain, leucine-rich containing family, pyrin domain-containing 3 (NLRP3) inflammasome in metastatic breast cancer. In vitro, we show that stimulation of THP-1 cells with conditioned media collected from MDA-MB-468 cells induced NLRP3 activation and increased *Pdcd1l1* expression. In vivo, mice deficient in NLRP3 orthotopically implanted with metastatic breast cancer cell line (E0771) showed significant reduction in tumor growth (*p* < 0.05) and increased survival (*p* < 0.01). Inhibition of NLRP3 with the small molecule OLT1177^®^ reduced expression of *Pdcd1l1* (*p* < 0.001), *Casp1* (*p* < 0.01) and *Il1b* (*p* < 0.01) in primary tumors. Furthermore, tumor-bearing mice receiving OLT1177^®^ showed reduced infiltration of myeloid-derived suppressor cells (MDSCs) (*p* < 0.001) and increased CD8^+^ T cells (*p* < 0.05) and NK cells (*p* < 0.05) in the TME. NLRP3 inhibition in addition to anti-PD-1 treatment significantly reduced tumor growth from the monotherapies (*p* < 0.05). These data define NLRP3 activation as a key driver of immune suppression in metastatic breast cancers. Furthermore, this study suggests NLRP3 as a valid target to increase efficacy of immunotherapy with checkpoint inhibitor in metastatic breast cancers.

## 1. Introduction

The proinflammatory cytokine interleukin-1β (IL-1β) is a potent driver of tumor progression by modulating tumor-promoting mechanisms such as angiogenesis, metastasis and immunosuppression [1,2,3]. IL-1β is originally produced as a biologically inactive precursor that requires processing by caspase-1 to be biologically active. This process is driven by activation of intracellular pattern recognition receptors termed NOD-like receptors. The nucleotide-binding domain, leucine-rich containing family, pyrin domain-containing 3 (NLRP3) inflammasome is a well-established mediator of IL-1β processing [4]. NLRP3 activation occurs predominantly in antigen-presenting cells such as macrophages, monocytes, neutrophils and dendritic cells, all of which comprise varying levels of infiltration in solid tumors [5].

Recently, Wu et al. demonstrated that IL-1β production in primary breast tumor biopsies positively correlated with disease severity [6]. Additionally, the authors demonstrated infiltrating CD11c^+^ myeloid cells as the source of IL-1β. Consistently, in an experimental model of triple-negative breast cancer, IL-1β deficient mice revealed significantly reduced levels of macrophages in the primary tumors, which were associated with limited tumor growth and an increase in infiltrating CD8^+^ T cells [3]. Taken together, these studies reveal a detrimental role of IL-1β in breast cancer progression and a prominent role of myeloid cell-derived IL-1β production. Therefore, further studies elucidating the mechanism(s) driving IL-1β production are warranted.

Increased expression of tumor-NLRP3 inflammasome has recently been described in various experimental models of solid tumors [7,8]. For example, in melanoma, it has been shown that tumor-NLRP3 activation drives chronic inflammation, resulting in anti-tumor immunity dysfunction [9,10,11]. Thus, these recent data suggest a tumor-promoting effect of NLRP3; however, its activation and role in breast cancer remain poorly defined.

Mechanistically, activation of NLRP3 promotes chronic inflammatory conditions through IL-1β induced expansion and activation of myeloid-derived suppressor cells (MDSCs). MDSCs are developmentally stunted neutrophils and monocytes induced under chronic inflammatory conditions. MDSCs exhibit potent immunosuppressive characteristics through the expression of immune modulator receptors such as PD-L1 or secreted molecules such as TGFβ or IL-10, ultimately suppressing T and NK cell activity [12,13]. In breast cancers, MDSC populations are expanded through increased signaling by cytokines and chemokines such as IL-6, G-CSF and CCL2 [14,15,16]. Importantly, IL-1β is a potent inducer of these cytokines, suggesting a possible mechanism for IL-1β mediated immunosuppression [17,18,19].

Recently, NLRP3 activity in melanoma was shown to upregulate PD-L1 in both tumor cells and MDSCs through tumor-intrinsic and tumor-host signaling pathways [9,11]. Consistently, a recent report using a 4T1 murine model of breast cancer showed addition of anti-PD-1 to anti-IL-1β, resulting in complete tumor abrogation through alterations of immunosuppressive cells [3]. To date, clinical studies assessing the efficacy of anti-PD-1 in metastatic breast cancers have shown overall response rates (ORR) only ranging from 5.3–18.5% [20,21]. Comparatively, the ORR to anti-PD-1 therapy in melanoma is 40% [22]. Therefore, further insights into the factors dictating whether breast cancers respond to checkpoint inhibition are needed. Considering the reported IL-1β signal reported in breast cancer, we sought to investigate whether NLRP3 activation is involved in the production of IL-1β in breast cancer and whether its inhibition would reduce the increased IL-1β signal and enhance anti-PD-1 therapy.

## 2. Results

### 2.1. NLRP3 Promotes Breast Cancer Progression

**Figure 1:**
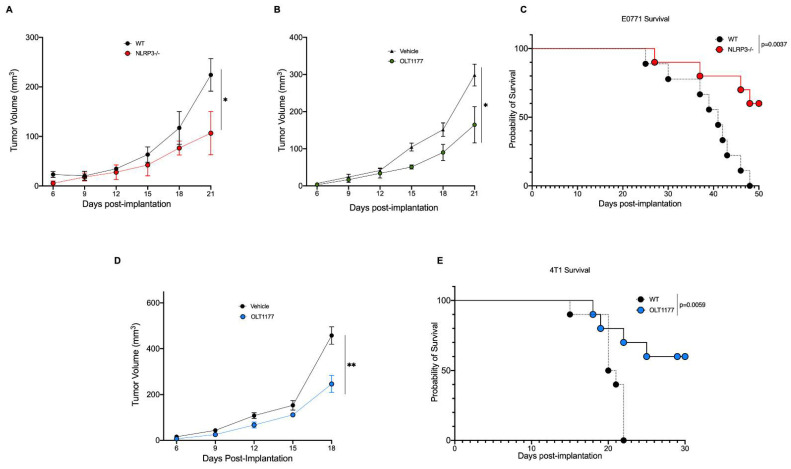
Genetic and pharmacologic inhibition of NLRP3 reduces breast cancer progression. (**A**) E0771 tumor growth in WT or *nlrp3^−/−^* mice (*n* = 3). (**B**) E0771 tumor growth in mice fed standard or OLT1177 diet (*n* = 6). (**C**) E0771 survival curve in WT or *nlrp3*^−/−^ (*n* = 10). (**D**) 4T1 tumor growth curve in mice fed standard or OLT1177 diet (*n* = 5). (**E**) 4T1 survival curve in mice fed standard or OLT1177 diet (*n* = 10). ** *p* < 0.01, * *p* < 0.05.

To assess the role of NLRP3 in breast cancer in vivo, the murine metastatic medullary breast adenocarcinoma cell line, E0771, was orthotopically implanted onto the mammary fat pad of wild type (WT) and NLRP3-deficient mice (*nlrp3*^−/−^). As shown in Figure 1A, tumor growth was significantly stunted in *nlrp3*^−/−^ mice compared to WT (*p* < 0.05). Next, we assessed whether pharmacologic inhibition of NLRP3 by the orally active NLRP3 inhibitor, OLT1177, would elicit similar effects. OLT1177 was administrated using drug-enriched diet (7.5 g/kg of food), and a matching standard diet was used as control, as previously described [23]. Mice were implanted with E0771 cells and 3 days post-implantation, mice were continued on standard diet or started on OLT1177 diet. Tumor-bearing mice on OLT1177 diet revealed significantly decreased tumor growth (Figure 1B) (*p* < 0.05), similarly to what we observed using the *nlrp3*^−/−^ mice (Figure 1A). Survival curve analysis for E0771 tumor-bearing mice was performed on WT and *nlrp3*^−/−^ mice. Mice deficient in NLRP3 showed markedly increased survival compared to WT mice (Figure 1C) (*p* = 0.0037). To confirm that the effect observed with NLRP3 inhibition was not cell line specific, the murine triple-negative breast cancer cell line 4T1 was also assessed. Mice were injected and started on OLT1177 diet as described above. As depicted in Figure 1D, mice fed OLT1177 revealed significantly slowed tumor growth compared to mice fed standard diet (*p* < 0.01). Additionally, 4T1 survival curve analysis was performed, revealing significantly improved survival in mice fed OLT1177 diet compared to mice fed standard diet (Figure 1E) (*p* = 0.0059). We next assessed whether OLT1177 altered cell proliferation in the murine cell lines 4T1 and E0771 as well as the human metastatic mammary adenocarcinoma line MDA-MB-468. No effect of OLT1177 on proliferation was observed, suggesting in vivo tumor reductions were not due to OLT1177 effects on tumor cell growth (Supplementary Figure S1). Overall, these data indicate a tumor-promoting role for host-NLRP3 in breast cancer.

### 2.2. Breast Cancer Secretome Induces Myeloid Expression of PD-L1 and NLRP3 Components

**Figure 2:**
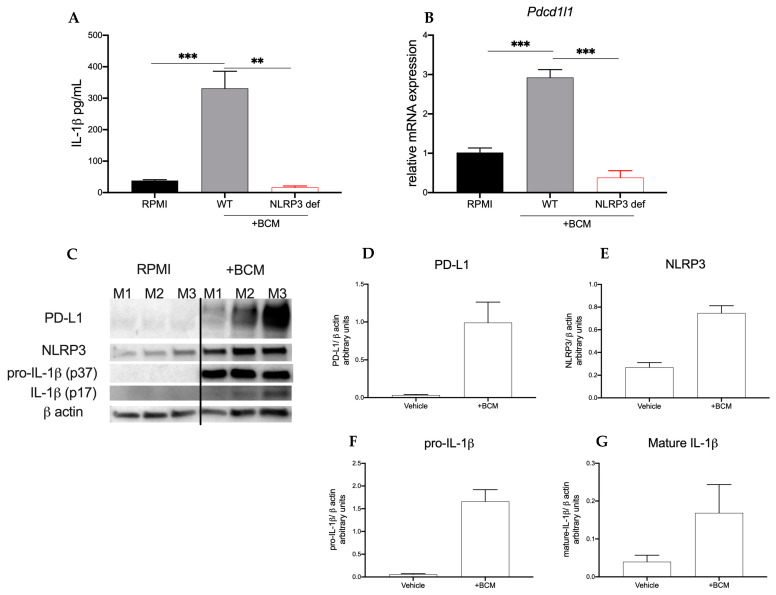
Breast cancer cells induce myeloid NLRP3 and PD-L1. (**A**,**B**) THP-1 or THP-1-NLRP3def cells were stimulated with MDA-MB-468-conditioned media (+BCM). (**A**) Mean ± SEM IL-1β production from THP-1 or THP-1-NLRP3def cells left unstimulated (RPMI) or treated with conditioned media after 48 h (*n* = 3). (**B**) Mean ± SEM of relative gene expression of *Pdcd1l1* from cells described in (**A**,**B**) or stimulated with (+BCM) and treated with OLT1177 (*n* = 3). (**C**–**G**) Bone marrow adherent cells stimulated with 4T1-conditioned media (+BCM). (**C**) Representative Western blot images from (**C**–**G**); mean ± SEM of PD-L1/β-actin ratio (**D**), NLRP3/β-actin ratio (**E**), pro-IL-1β/β-actin ratio (**F**) and mature IL-1β/β-actin ratio (**G**) from cells described in (**C**–**G**) (*n* = 3). *** *p* < 0.001, ** *p* < 0.01.

Increased peripheral monocytes in patients with breast cancer are associated with decreased overall survival [24]. Therefore, considering our observation that in NLRP3-deficient mice tumor progression was stunted, we hypothesized that the infiltrating myeloid cells were propagating pro-tumor signaling. To start, we used the human monocyte cell line, THP-1. Both native (WT) and NLRP3-deficient THP-1 (THP-1defNLRP3) cells were stimulated with breast cancer-conditioned media (BCM) obtained from MDA-MB-468 cells to study the effect of the tumor secretome on myeloid cells. As shown in Figure 2A, THP-1 cells stimulated with BCM showed significantly increased IL-1β (*p* < 0.001) secretion compared to the cells in control (Figure 2A). THP-1defNLRP3 cells showed reduced IL-1β (*p* < 0.01) secretion compared to native THP-1 cells stimulated with BCM (Figure 2A), suggesting an NLRP3-dependent IL-1β production. To determine the effect of NLRP3 activation on immunosuppressive markers, we assessed *Pdcd1l1* mRNA levels following BCM stimulation. As shown in Figure 2B, native THP-1 cells significantly upregulate *Pdcd1l1* expression after stimulation with BCM (*p* < 0.05). This increase was not observed in the THP-1defNLRP3 cells (Figure 2B) (*p* < 0.001). Lastly, we sought to assess whether pharmacologic inhibition of NLRP3 with OLT1177 would exhibit effects similar to those observed with the genetically altered THP-1 cells. THP-1 cells were stimulated with BCM as described above, with or without OLT1177 (10 μM), and *Pdcd1l1* expression was measured. Accordingly, cells treated with OLT1177 showed no increase in *Pdcd1l1* expression following stimulation with BCM (Figure 2B) (*p* < 0.01). Next, we assessed whether soluble factors secreted from 4T1 cells affected IL-1 signaling in the myeloid compartment. Bone marrow cells were harvested from WT mice; the adherent fraction was then stimulated with 4T1-conditioned media (BCM) or RPMI for 3 days, and Western blot analysis was performed on NLRP3, IL-1β and PD-L1. As shown in Figure 2C–G, BCM (right) upregulated expression of PD-L1, NLRP3, pro-IL-1β (p37) and mature IL-1β (p17) when compared to the same cells in control (left) (Figure 2F). These data suggest that breast cancer cells secrete soluble factors that act on myeloid cells activating the NLRP3 inflammasome, which in turn upregulate immunosuppressive markers.

### 2.3. NLRP3 Inhibition Reduces Immunosuppression in TME

**Figure 3:**
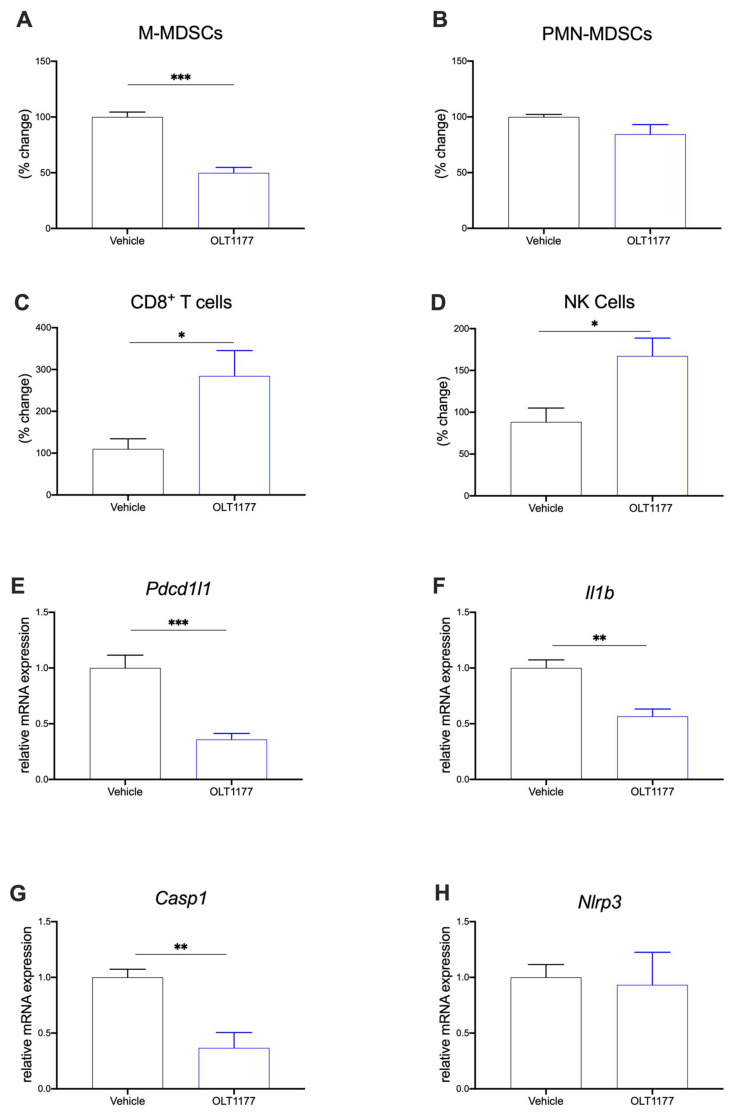
NLRP3 drives immunosuppressive TME. (**A**–**D**) Flow cytometry analysis of primary tumors in mice fed standard or OLT1177 diet. (**A**) Levels of M-MDSC in primary tumors of mice fed standard or OLT1177 diet (*n* = 5). (**B**) Levels of PMN-MDSC in primary tumors of mice fed standard or OLT1177 diet (*n* = 5). (**C**) Levels of CD8^+^ T cells in primary tumors of mice fed standard or OLT1177 diet (*n* = 5). (**D**) Levels of NK cells in primary tumors of mice fed standard or OLT1177 diet (*n* = 5). (**E**–**G**) Mean ± SEM of relative mRNA expression of *Pdcd1l1* (E), *Il1β* (**F**), *Casp1* (**G**) and *Nlrp3* (**H**) from primary 4T1 tumors in mice fed standard or OLT1177 diet (*n* = 6). *** *p* < 0.001, ** *p* < 0.01, * *p* < 0.05.

To investigate the downstream effect of NLRP3 inhibition on the TME composition, flow cytometry and gene expression analyses were performed on primary tumors. Mice were implanted with 4T1 tumors and fed OLT1177 or standard diets as previously described. MDSCs (both M-MDSCs CD11b^+^Ly6G^−^Ly6C^hi^ and PMN-MDSCs CD11b^+^Ly6G^+^Ly6C^lo^) and T cell and NK cell populations were assessed in the TME. Mice fed OLT1177 diet revealed significantly decreased M-MDSCs compared to mice on the standard diet (Figure 3A) (*p* < 0.001). No significant changes were observed in PMN-MDSC (Figure 3B). Furthermore, an increase in CD8^+^ T cells and NK cells was observed in mice fed OLT1177 diet when compared to the tumor-bearing mice fed standard diet (Figure 3C,D) (*p* < 0.05).

Considering the effects of BCM on protein expression in bone marrow adherent cells described above, we also assessed gene expression in primary tumors. 4T1 tumors from mice described above were assessed for *Pdcd1l1*, *Il1b*, *Casp1*, and *Nlrp3* gene expression. Mice fed OLT1177 diet expressed significantly less *Pdcd1l1* (Figure 3E), *Il1b* (Figure 3F) and *Casp1* (Figure 3G) compared to mice fed standard diet. As expected by the mechanism of OLT1177, no changes in *Nlrp3* gene expression were observed (Figure 3H) [25]. Similar results were observed in in the E0771 model. Mice fed OLT1177 again showed decreased gene expression of *Pdcd1l1*, *Il1b* and *Casp1* with no changes observed in *Nlrp3* (Supplementary Figure S2A–D) (*p* < 0.05).

Lastly, we assessed the role of host-NLRP3 on the expression of PD-L1 in the TME using flow cytometry. E0771 cells were orthotopically implanted into wild-type (WT) or NLRP3-deficient mice (*nlrp3*^−/−^). As shown in Supplementary Figure S3A, no changes in PD-L1 expression were observed in total live cell populations in tumors from WT or *nlrp3^−/−^* mice. We then assessed PD-L1 expression on myeloid populations from the TME. Dendritic cells (CD11b^+^Ly6C^−^CD64^−^MHCII^+^) from WT or *nlrp3*^−/−^ mice showed no difference in PD-L1 (Supplementary Figure S3B); however, macrophages (CD11b^+^Ly6G^−^CD64^+^MHCII^−/+^) from *nlrp3^−/−^* mice expressed significantly less PD-L1 compared to WT mice (Supplementary Figure S3C) (*p* *<* 0.05). We concluded that activation of NLRP3 was driving the recruitment of immunosuppressive cell populations, which resulted in a reduction in anti-tumor immunity and ultimately led to tumor progression.

### 2.4. NLRP3 Inhibition Enhances Anti-PD-1 Efficacy

**Figure 4:**
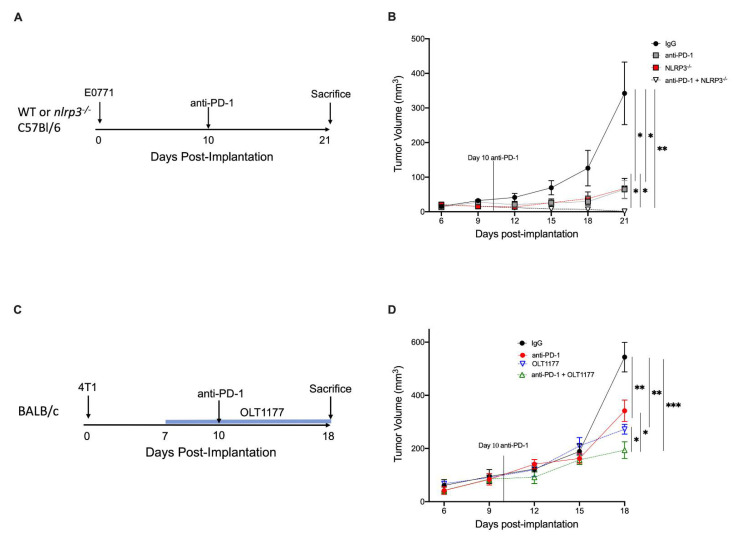
NLRP3 inhibition enhances anti-PD-1 efficacy. (**A**) Experimental design; (**B**). (**B**) E0771 tumor growth in WT and *nlrp3^−/−^* mice treated with anti-PD-1 or IgG (*n* = 4). (**C**) Experimental design (**D**). (**D**) 4T1 tumor growth in mice fed standard or OLT1177 diet and treated with anti-PD-1 or IgG (*n* = 5). *** *p* < 0.001, ** *p* < 0.01, * *p* < 0.05.

MDSC recruitment to the TME leads to a potent immunosuppressive environment that can account for the limited efficacy of immunotherapy in cancer [26,27]. Considering the reduced PD-L1 expression and M-MDSC infiltration following NLRP3 inhibition, we investigated the effect of anti-PD-1 therapy on mice that were deficient for NLRP3. WT or *nlrp3^−/−^* mice were implanted with E0771 tumors, and 10 days after the implantation, a single dose of anti-PD-1 (8 mg/kg) or a matching dose of IgG (8 mg/kg) was administered intraperitoneally (Figure 4A). Consistent with our previous findings, Figure 4B reveals *nlrp3^−/−^* mice treated with control IgG exhibited reduced tumor growth compared with WT mice treated with control IgG (*p* < 0.05). Tumor growth was significantly reduced in WT mice receiving anti-PD-1 compared to the WT IgG control (*p* < 0.05). Notably, treatment with anti-PD-1 in *nlrp3^−/−^* mice resulted in near complete tumor abrogation at day 21 (*p* < 0.01) with significant reduction compared to monotherapy anti-PD-1 or *nlrp3^−/−^* mice alone (*p* < 0.05) (Figure 4B).

Next, we evaluated the therapeutic potential of adding OLT1177 to anti-PD-1. Mice were implanted with 4T1 tumors; on day 7 post-implantation, mice were continued on standard diet or started on OLT1177 diet. On day 10, IgG or anti-PD-1 was administered (Figure 4C). As shown in Figure 4D, mice receiving either OLT1177 or anti-PD-1 revealed significant reductions in tumor growth compared to IgG control (50% and 38%, respectively) (*p* < 0.01). Combination of the therapies further reduced tumor growth compared to anti-PD-1 (44%) or OLT1177 (29%) alone (*p* < 0.05) (Figure 4D).

Consistent with the observations in the 4T1 model, addition of OLT1177 to anti-PD-1 showed similar efficacy in the E0771 model. As shown in Supplementary Figure S4, both OLT1177 and anti-PD-1 monotherapies showed significant reductions in tumor growth compared to IgG control (*p* < 0.05). E0771 tumor-bearing mice receiving both OLT1177 and anti-PD-1 showed markedly stunted tumor growth compared to IgG (*p* < 0.001) (Supplementary Figure S4). Combination of the therapies further reduced tumor growth compared to either monotherapy alone (*p* < 0.05) (Supplementary Figure S3). These data propose NLRP3 inhibition as a strategy for increasing anti-PD-1 efficacy in metastatic breast cancers.

## 3. Discussion

In the current study, we demonstrated that metastatic breast cancer cells induce activation of NLRP3 in myeloid cells, ultimately resulting in an immunosuppressive TME. Moreover, we proposed OLT1177 as a promising therapeutic adjuvant to anti-PD-1 therapy in metastatic breast cancers.

Elevated IL-1β correlates with disease severity and poor outcomes in advanced-stage breast cancers [6]. In an ex vivo study of 239 biopsies, IL-1β production was measured using tumor biopsies stimulated with PMA and correlated to disease severity [6]. Interestingly, a recent study suggested that soluble CD44 released by triple-negative breast cancer cells promotes tumor progression by triggering macrophage IL-1β production [28]. The authors found inflammasome-dependent IL-1β secretion showing ASC oligomerization, although the specific role of NLRP3 or other NOD proteins was not investigated. Here, we expanded upon those findings by using genetic and pharmacological NLRP3 inhibition to elucidate NLRP3 as the predominate inflammasome sensor responsible for breast tumor-induced IL-1β production in infiltrating myeloid cells. Although other sources of NLRP3 are possible, this study showed a clear induction of IL-1β production in myeloid cells by breast tumor cells [29]. Our data highlight NLRP3 as a key molecular driver of IL-1β production as well as outline targeted therapeutic options.

NLRP3 activation in cancer has been attributed to response to chemotherapy in triple-negative breast cancer and oral squamous cell carcinoma [30,31]. In this study, we showed that IL-1β production in breast cancers is driven by NLRP3 activation in myeloid cells at disease baseline. Notably, we also showed that pharmacological inhibition of NLRP3 with the inhibitor OLT1177 reduces IL-1β levels, ultimately reducing tumor progression.

Chronic IL-1β secretion promotes infiltration of immunosuppressive myeloid cells into the TME, which then act as potent suppressors of cytotoxic T cell and NK cell activity [3,32]. In breast cancers, MDSC levels correlate with poor clinical outcomes and increased metastasis [33,34,35]. In this study, we demonstrated that inhibition of NLRP3 inflammasome resulted in less M-MDSCs in the TME with subsequent increased CD8^+^ and NK cell infiltration. Of the main immunosuppressive characteristics of MDSCs, expression of PD-L1 presents itself as one of the most accessible targets for therapeutic intervention [36]. Here, tumor-bearing mice treated with OLT1177 showed less *Pdcd1l1* expression in primary tumors. These findings support a previous study by our group, which revealed that inhibition of NLRP3 signaling in mice implanted with B16 melanoma decreases *Pdcd1l1* gene expression in MDSCs [11].

Much has been reported on the expression of PD-L1 on tumor cells [37,38]. Canonically, IFNγ secreted by T cells induces PD-L1 expression in multiple tumor types [39,40,41]. Importantly, monocyte regulation of PD-L1 expression can be induced by non-IFNγ cytokines [42]. The data presented in this study represent a T cell-independent mechanism to PD-L1 induction in myeloid populations. Importantly, we showed how stimulation of bone marrow adherent cells with 4T1-conditioned media markedly increased PD-L1 expression. Furthermore, upon stimulation with MDA-MB-468 conditioned media, OLT1177 reduced *Pdcd1l1* expression in THP-1 cells back to unstimulated levels. In several solid tumors, it has been shown that the TME consists of high proportions of myeloid cells; therefore, consideration of this additional barrier to effective usage of checkpoint inhibitors remains pertinent.

Although immunotherapy with checkpoint inhibitors has revolutionized treatment for several cancers, the benefits of using checkpoint inhibition in triple-negative breast cancer are quite limited, representing an important unmet clinical need. In two single-arm studies assessing the efficacy of Pembrolizumab (anti-PD-1) in metastatic triple-negative breast cancer, the ORR was 18.5% in the phase 1b KEYNOTE-012 study and 5.3% in the phase 2 KEYNOTE-086 study [20,21]. Additionally, in the KETNOTE-119 study of Pembrolizumab versus investigator choice chemotherapy for metastatic triple-negative breast cancer, the ORR was 9.6% compared to 10.6% in the chemotherapy cohort [43]. Considering the marginal effect of immunotherapy and our observation that NLRP3 activation increased PD-L1 expression, we tested the efficacy of combining anti-PD-1 and NLRP3 inhibition. We showed how inhibition of NLRP3-dependent PD-L1 expression of myeloid cell origin increases the efficacy of anti-PD-1. This approach of increasing anti-PD-1 efficacy has been validated in pre-clinical experimental models in metastatic melanoma and renal cell carcinoma through modulating PD-L1 expression downstream of NLRP3 activation [11,44]. Thus, existing pre-clinical and clinical data suggest a prominent role for NLRP3 blockade in metastatic breast cancers, underscoring OLT1177 in combination with anti-PD-1 as a valid treatment option.

## 4. Materials and Methods

### 4.1. Mice

Animal protocols were approved by the University of Colorado Animal Care and Use Committee. Wild type female BALB/c, C57Bl/6 or *nlrp3^−/−^* (B6.129S6-Nlrp3^tm1Bhk^/J) mice were purchased from The Jackson Laboratory (Bar Harbor, ME, USA) (6–8 weeks old).

### 4.2. Cell Lines

Mammary carcinoma cells lines 4T1, E0771 and MDA-MB-468 were purchased from ATCC (Manassas, VA, USA), and native THP-1 and NLRP3-deificient THP-1 cells were purchased from InvivoGen (San Diego, CA, USA). 4T1 and MDA-MB-468 cells were cultured in DMEM (Corning, Corning, NY, USA) supplemented with 10% FBS, 100 units/mL penicillin and 0.1 mg/mL streptomycin. E0771 cells were cultured in RPMI (Corning) supplemented with 10% FBS, 1% HEPES, 100 units/mL penicillin and 0.1 mg/mL streptomycin. Cells were maintained in a humidified 5% CO_2_ atmosphere at 37 °C.

### 4.3. OLT1177^®^

OLT1177 was administrated in OLT1177-enriched diet at the concentration of 7.5 g of OLT1177 per kg of food. A matching diet lacking OLT1177 was used as control (standard diet). Both diets were purchased from Research Diets Inc (New Brunswick, NJ, USA). OLT1177 used in vitro was provided by Olatec Therapeutics.

### 4.4. Breast Cancer Conditioned Media Assays

Supernatants from MDA-MB-468 cells (BCM) were added to native THP-1 or NLRP3-deficient THP-1 cells (1 × 10^5^) (InvivoGen) and cultured in a 96-well plate and activated with 10 ug/mL of LPS (Sigma Aldrich, Burlington, MA, USA) for 3 h. BCM was then added at 1:2. Cells were incubated for 3 days before collection of the supernatants or cells for gene analysis. Bone marrow adherent cells were obtained from WT BALB/c female mice, and 5 million cells were plated into a 12-well plate with cell culture media. The next day the non-adherent fraction was removed, and cells were then treated with 4T1-conditioned media (1:3 conditioned media to RPMI) or normal media. On day 3, cells were lysed in RIPA buffer and prepared for Western blots.

### 4.5. In Vivo Tumor Model

Cell lines were trypsinized, and 2 × 10^5^ cells/mouse were suspended in Matrigel (Corning) and injected orthotopically into single mammary fat pad of BALB/c (for 4T1) or C57Bl/6 mice (for E0771). Tumor growth was assessed every 3 days using a caliper, and tumor volume was calculated as ½ (L × W × H). Mice were sacrificed after 15/18 (4T1) or 18/21 (E0771) days from tumor cell implantation. Mice were fed OLT1177-enriched diet or a standard food diet after tumor cell implantation. Survival curves were calculated with tumor volume > 500 mm^3^ set as end point with 5 × 10^4^ cells injected for E0771 and 2 × 10^5^ for 4T1.

### 4.6. Combination Therapy Model

4T1 or E0771 cells were implanted as described above. On day 7 after implantation, mice were started on the OLT1177 diet or continued on standard diet. At day 10, a neutralizing antibody against PD-1 (RMP1.14) (Catalog#BP0146) (8 mg/kg in 200 μL saline; BioXCell, Lebanon, NH, USA) or IgG (8 mg/kg in 200 μL saline; BioXCell) (Catalog#BP0089) was injected. Mice were sacrificed 21 days post tumor implantation.

### 4.7. MTS Proliferation Assay

Cell proliferation was determined according to manufacturer’s recommendations (Abcam, Cambridge, MA, USA). 4T1, E0771 and MDA-MB-468 cells were cultured as described above in the presence and absence of OLT1177 for 24 h, and absorbance was determined at 490 nm.

### 4.8. Flow Cytometry

Tumors were harvested and homogenized using 40 μm cell strainers. Tumor cells were then washed and resuspended in PBS at 5 × 10^6^/mL. Single-cell suspensions were run on BD FACSCelesta Cell Analyzer (BD Biosciences, Franklin Lakes, NJ, USA) and analyzed on FlowJo Software (FlowJo, Ashland, OR, USA). Single-cell suspensions were stained using antibody list anti-CD45 Pe/Cy7 (BioLegend, San Diego, CA, USA), anti-CD11b BV785 (BioLegend), anti-CD3 Alexa Fluor700 (eBioscience, San Diego, CA, USA), anti-Ly6G PacBlue (BioLegend), anti-Ly6C PerCp/Cy5.5 (Biolegend), anti-CD-161 APC (BioLegend), anti-CD335 BV650 (BioLegend), anti-CD8 Alexa Fluor 488 (BioLegend), anti-CD11b APC Cy7 (BioLegend), anti-CD64 BV605 (BioLegend), anti-MHCII AlexaFluor488 (BioLegend) and anti-PDL1 APC (Biolegend).

### 4.9. Cytokine Measurements

Cytokines were measured by specific DuoSet ELISAs according to the manufacturer’s instructions (R&D Systems, Minneapolis, MN, USA). A Bio-tek EL × 800 microplate reader was used to measure optical density (Agilent, Santa Clara, CA, USA).

### 4.10. Gene Expression

Primary tumors were collected as described above. RNA was then isolated using Trizol (Thermo Fisher Scientific, Waltham, MA, USA) and synthesized in cDNA using SuperScript III First-Strand (Thermo Fisher Scientific). Quantitative PCR (qPCR) was performed on cDNA using Power SYBR Green PCR master mix (Thermo Fisher Scientific) on a Biorad CFX96 Real time system. Cycles were set per manufacturer’s suggestion. Gene expression was assessed for the following mRNAs: *Pdcd1l1* (forward 5′-GCTCCAAAGGACTTGTACGTG-3′ and reverse 5′-TGATCTGAAGGGCAGCATTTC-3′), *Il1b* (forward 5′-CTAAACAGATGAAGTGCTCCTTCC-3′ and reverse 5′-CACATAAGCCTCGTTATCCCA-3′), *Casp1* (forward 5′-AAGTCGGCAGAGATTTATCGA-3′ and reverse 5′-GATGTCAACCTCAGCTCCAG-3′), *Nlrp3* (forward 5′-GAATCTCACGCACCTTTACC-3′ and reverse 5′-GCAGTTGTCTAATTCCAACACC-3′). Relative gene expression was normalized with *18s* for mice and *Gapdh* for human: *18s* (forward 5′-GTAACCCGTTGAACCCCATT-3′ and reverse 5′-CCATCCAATCGGTAGTAGCG-3′), *Gapdh* (forward 5′-TGTGGGCATCAATGGATTTGG-3′ and reverse 5′-ACACCATGTATTCCGGGTCAAT-3′).

### 4.11. Western Blotting

Bone marrow adherent cells were cultured as previously described. All cells were lysed in RIPA buffer (Sigma, St. Louis, MS, USA) supplemented with protease inhibitors (Roche, Indianapolis, IN, USA), centrifuged at 13,000× *g* for 20 min at 4 °C, and the supernatants were obtained. Protein concentration was determined in the clarified supernatant using Bio-Rad protein assay (Bio-Rad Laboratories, Hercules, CA, USA). Proteins were electrophoresed on Mini-PROTEAN TGX 4−20% gels (Bio-Rad Laboratories) and transferred to nitrocellulose 0.2 mm (GE Water & Process Technologies, Feasterville-Trevose, PA, USA). Membranes were blocked in 5% rehydrated non-fat milk in PBS-Tween 0.5% for 1 h at room temperature. Primary antibodies for PD-L1 (1:250) (Catalog#AF1019) (R&D Systems), NLRP3 (1:1000) (Catalog#AG-20B-0014-C100) (Adipogen, San Diego, CA, USA), and IL-1β (1:250) (Catalog#AF-401-NA) (R&D Systems) were used in combination with peroxidase-conjugated secondary antibodies. A primary antibody against β-actin (Santa Cruz Biotechnology, Dallas, TX, USA) was used to assess protein loading. Chemiluminescence was captured using Bio-Rad Chemidoc MP Imaging System (Bio-Rad Laboratories). Images were quantified using ImageJ (U.S. National Institutes of Health, Bethesda, MD, USA).

### 4.12. Statistical Analysis

Statistical significance of differences was evaluated with a two-tailed Student’s *t* test with * *p* < 0.05, ** *p* < 0.01 and *** *p* < 0.001 and log-rank (Mantel–Cox and Gehan–Breslow–Wilcoxon) tests for survival analysis using Prism version 7.0 software (GraphPad Software, La Jolla, CA, USA).

## 5. Conclusions

In conclusion, we revealed that breast cancer-induced NLRP3 activation results in the expression of PD-L1 in myeloid cells and proposed NLRP3 inhibition as a promising therapeutic approach to overcome anti-PD-1 resistance

## Data Availability

Data is contained within the article and supplementary material.

## Supplementary Materials

The following supporting information can be downloaded at: https://www.mdpi.com/article/10.3390/ph15050574/s1, Figure S1: Effect on OLT1177 on breast cancer cell growth in vitro; Figure S2: OLT1177 reduces immunosuppression-associated mRNA levels in E0771 TME. (A–D) Mean ± SEM of relative mRNA expression of (A) *Pdcd1l1*, (B) *Il1b*, (C) *Casp1* and (D) *Nlrp3*. * *p* < 0.05; Figure S3: Flow cytometry analysis of E0771 tumors from WT or *nlrp3^−/−^* mice. (A) PD-L1 expression on total live cells from TME, (B) dendritic cell expression of PD-L1, (C) macrophage expression of PD-L1. * *p* < 0.05; Figure S4: Synergistic effect of OLT1177 in combination with anti-PD-1 in E0771 model. ** *p* < 0.001, * *p* < 0.05.
