# Supplementary material for: Activation of Host-NLRP3 Inflammasome in Myeloid Cells Dictates Response to Anti-PD-1 Therapy in Metastatic Breast Cancers
Source: Pharmaceuticals (Basel). 2022 May 4;15(5):574. doi: 10.3390/ph15050574 (PMC9144656; doi:10.3390/ph15050574)

Supplemental Figure S1: Effect on OLT1177 on breast cancer cell growth in vitro.

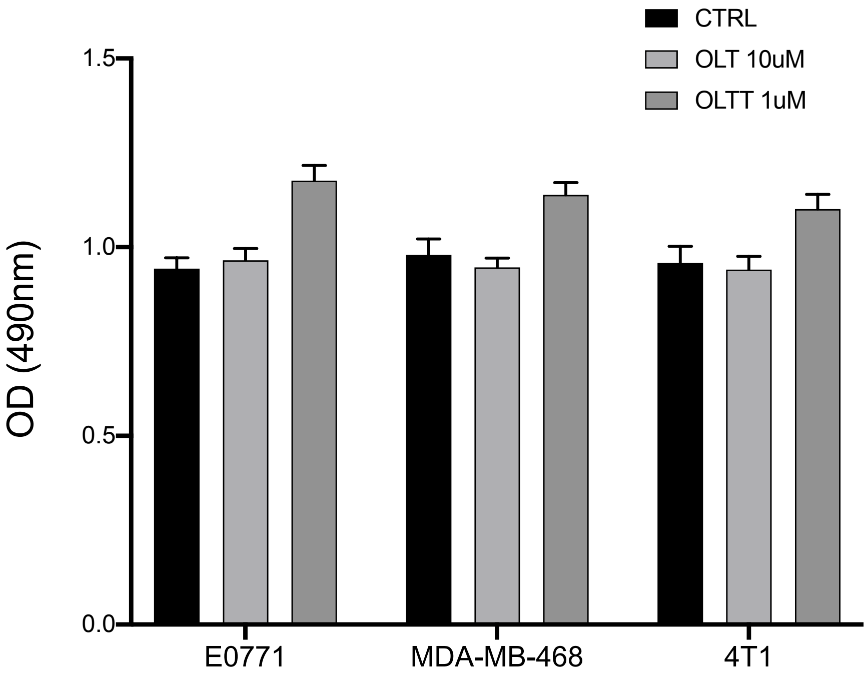

Supplemental Figure S2: OLT1177 reduces immunosuppression-associated mRNA levels in E0771 TME. (A–D) Mean ± SEM of relative mRNA expression of (A)*Pdcd1l1*, (B) *Il1b*, (C) *Casp1* and (D) *Nlrp3*. * $p < 0.05$.

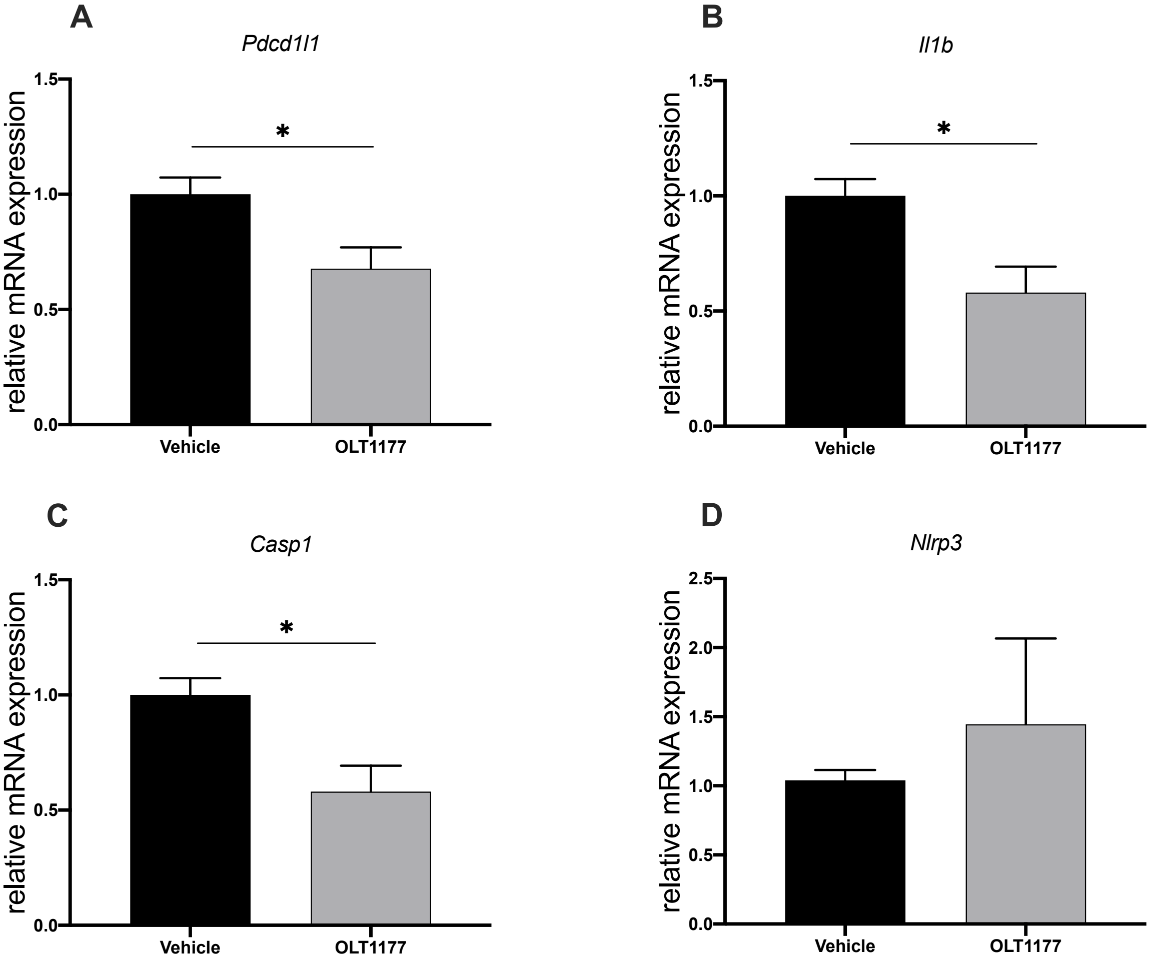

Supplemental Figure S3: Flow cytometry analysis of E0771 tumors from WT or *nlrp3*$^{-/-}$ mice. (A) PD-L1 expression on total live cells from TME, (B) dendritic cell expression of PD-L1, (C) macrophage expression of PD-L1. * $p < 0.05$.

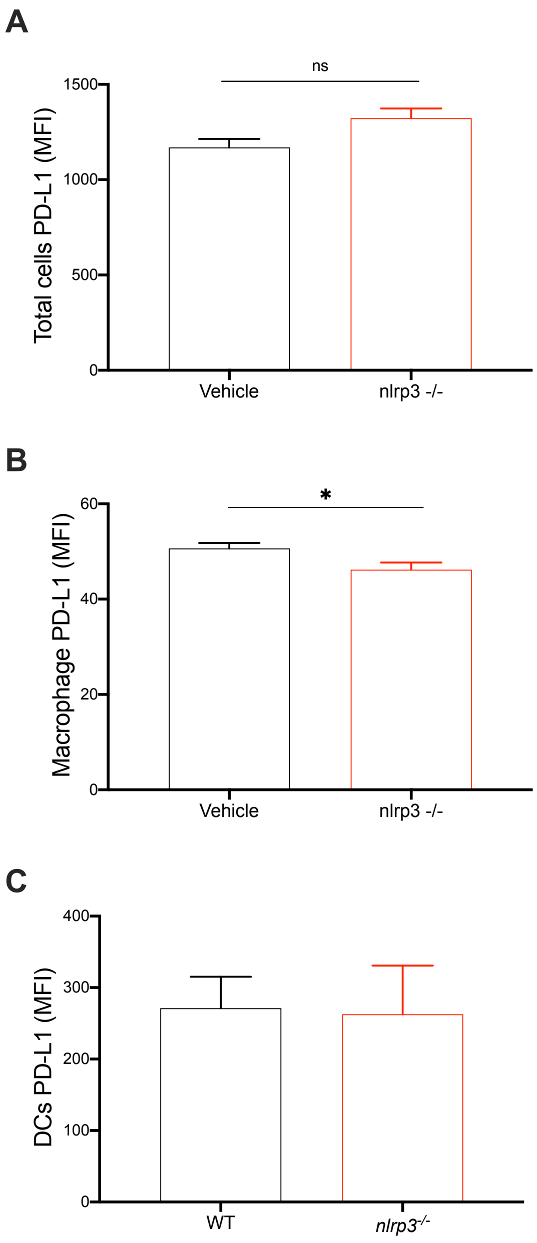

Supplemental Figure S4: Synergistic effect of OLT1177 in combination with anti-PD-1 in E0771 model. ** $p < 0.001$, * $p < 0.05$.

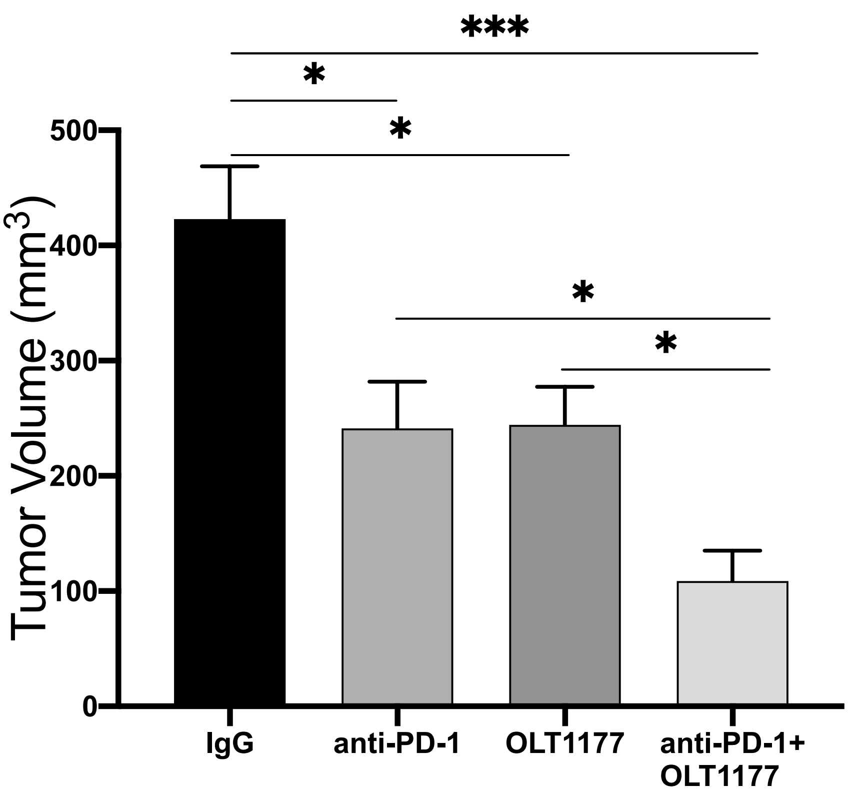

Supplement: Supplementary file 1 [file pharmaceuticals-15-00574-s001.zip › pharmaceuticals-1628444-supplementary.pdf]
